# Comparative Life Cycle Assessment of SLS and mFFF Additive Manufacturing Techniques for the Production of a Metal Specimen

**DOI:** 10.3390/ma17010078

**Published:** 2023-12-23

**Authors:** Andrea Presciutti, Elisa Gebennini, Federica Liberti, Francesca Nanni, Mario Bragaglia

**Affiliations:** 1 Faculty of Technological and Innovation Sciences, University Mercatorum, Piazza E. Mattei Rome, 00186 Roma, Italy; 2 Department of Enterprise Engineering “Mario Lucertini”, University of Rome “Tor Vergata” and INSTM RU Roma-Tor Vergata, Via del Politecnico 1, 00133 Rome, Italy

**Keywords:** LCA, 3D printing, MAM, metal Fused Filament Fabrication, Selective Laser Sintering

## Abstract

This work is part of a research project aimed at developing a bio-based binder, composed mainly of polylactic acid (PLA), to produce Ti6Al4V feedstock suitable for use in MAM (Metal Additive Manufacturing) via mFFF (metal Fused Filament Fabrication), in order to manufacture a titanium alloy specimen. While in Bragaglia et al. the mechanical characteristics of this sample were analyzed, the aim used of this study is to compare the mentioned mFFF process with one of the most used MAM processes in aerospace applications, known as Selective Laser Sintering (SLS), based on the Life Cycle Assessment (LCA) method. Despite the excellent properties of the products manufactured via SLS, this 3D printing technology involves high upfront capital costs while mFFF is a cheaper process. Moreover, the mFFF process has the advantage of potentially being exported for production in microgravity or weightless environments for in-space use. Nevertheless, most scientific literature shows comparisons of the Fused Filament Fabrication (FFF) printing stage with other AM technologies, and there are no comparative LCA “Candle to Gate” studies with mFFF processes to manufacture the same metal sample. Therefore, both MAM processes are analyzed with the LCA “Candle to Gate” method, from the extraction of raw materials to the production of the finished titanium alloy sample. The main results demonstrate a higher impact (+50%) process for mFFF and higher electrical energy consumption (7.31 kWh) compared to SLS (0.32 kWh). After power consumption, the use of titanium becomes the main contributor of Global Warming Potential (GWP) and Abiotic Depletion Potential (ADP) for both processes. Finally, an alternative scenario is evaluated in which the electrical energy is exclusively generated through photovoltaics. In this case, the results show how the mFFF process develops a more sustainable outcome than SLS.

## 1. Introduction

### 1.1. Eco-Friendly Additive Manufacturing

Additive Manufacturing (AM), also known as 3D printing, is a manufacturing process that has gained significant attention and popularity in recent years. It involves creating three-dimensional objects by adding material layer by layer, unlike traditional subtractive manufacturing methods that involve cutting or shaping material from a solid block [1]. The process of additive manufacturing begins with a digital design file created using computer-aided design (CAD) software or obtained through 3D scanning [2]. This design is then sent to a 3D printer, which interprets the file and builds the object using various additive techniques categorized by ISO/ASTM52900 [3] into seven different main groups: Binder Jetting (BJ), Directed Energy Deposition (DED), Material Extrusion (ME), Material Jetting (MJ), Powder Bed Fusion (PBF), Sheet Lamination, and VAT Polymerization [3]. Among AM technologies, one of the processes that is gaining relevance based on its ability to manufacture functional parts is Metal AM (MAM). The most important MAM advantage is that it enables the production of highly complex geometries that would be difficult or impossible to create using traditional manufacturing methods. It also allows rapid prototyping, reducing the time and cost associated with traditional methods and tools [4].

MAM has found applications across various industries, including aerospace, automotive, healthcare, consumer products, and more. It has been used to produce functional prototypes, end-use parts, architectural models, medical implants, and even food [5]. In the medical field, MAM has achieved success in the fabrication of surgical titanium implants [6]. In aviation, the GE9X engine for the Boeing 777 now incorporates a heat exchanger assembly comprising a single component that is 25% cheaper and 40% lighter [7]. SpaceX has reduced production time and weight by 40% for its Super Draco and Raptor engines [8]. NASA is also set to replace Space Shuttle main engine components with MAM parts to decrease production time and weight [9]. Nevertheless, it is important to highlight that unlike well-established metalworking techniques, there is still a lack of research understanding in the MAM sector regarding the relationship between processing, structure, and properties [10].

According to Suwanpreecha et al. [11], the most widely used MAM processes for the production of metal components are: Powder Bed Fusion (PBF), Direct Energy Deposition (DED), and Material Extrusion Additive Manufacturing (MEAM) [10]. The PBF process has the advantage of good printing resolution, but relatively slow deposition. The DED benefits from its relatively fast printing (high build rate), but achieves only a near-net shape (coarse printing resolution). The MEAM process has a balanced performance with a relatively sufficient resolution, an adequate printing time, and a reasonably low operation cost [12].

In several studies, additional terms are also used to indicate the MEAM process, such as: Material Extrusion (MEX), SDS (Shaping, Debinding, and Sintering), [13] or Fused Filament Fabrication (FFF) [4,10,11], usually called Fused Deposition Modelling (FDM) [11,14]; the latter is a relatively new technique. However, since FFF is related to a metal specimen, the examined MEX procedure is, therefore, referred to as metal FFF (mFFF) [12].

Kokare et al. [1] observed that MEAM technologies have been studied extensively compared to other AM technologies. In particular, the adoption of FFF has experienced rapid growth [15]. This trend reflects the industry’s aspiration to enhance its environmental sustainability and embrace a low-carbon approach for long-term development. FFF has promptly succeeded or complemented traditional processes, particularly in the realm of single-piece and low-volume processing within the plastics sector.

According to Ruschi et al. [16], as environmental constraints increasingly play a significant role in manufacturing technologies, the number of studies on environmental impacts of AM is expected to grow considerably in the coming years.

As reported by Faludi and colleagues [17], this statement can be found in numerous publications that aim to verify the environmental sustainability of the AM process. As Le Bourhis [18] argues, the different additive manufacturing processes do not have the same environmental impacts. The review by Khalid and Peng [19] discusses that in the Scopus, Web of Science, and Science Direct databases, between 2011 and 2021, over 300 articles related to the analysis of the sustainability of AM processes are cited. Upon researching AM sustainability, Gao et al. [4] stated that Life Cycle Assessment (LCA) is an effective tool for sustainability analysis and could help to further evaluate how metal AM contributes to global sustainability.

### 1.2. Life Cycle Assessment of MAM

The LCA analysis method provides a comprehensive view of the environmental consequences of different process choices and can inform decision-making processes aimed at reducing environmental burdens. It can help identify areas in need of improvement, guide eco-design strategies, and support the development of sustainable practices and policies [20].

**Figure 1:**
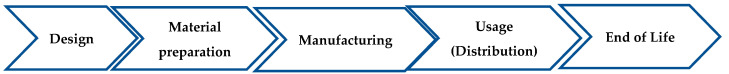
Diagram of the elementary stages of the manufacturing process [21].

**Figure 2:**
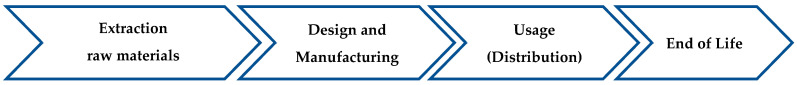
Diagram of the elementary stages of the AM of metal parts [1,4].

The most common model used in the LCA method is defined as “Cradle-to-Grave” where the manufacturing process is divided into five stages (shown in Figure 1) [21,22]. Figure 2 illustrates, more specifically, the “Cradle-to-Grave” LCA method of MAM; this process begins with the extraction of raw materials and powder production [4].

Instead, the (LCA) “Cradle-to-Gate” measures a product’s environmental impact up to the point where it leaves the factory gate. This means the environmental impact results do not include the product’s use by customers or its end-of-life processes.

After having analyzed the most recent review articles [16,18,23], many different approaches and choices in structuring an LCA of AM were revealed. Each choice (such as the boundaries of the system or renewable energy use) has the potential to influence the outcome of the study [24]. Therefore, to correctly interpret potential environmental impacts measured through LCAs, it is always necessary to understand the methodological reasoning behind the assessment.

With the rapid advancement of 3D printing technology and the growing prevalence of FFF in industrial production, researchers have progressively undertaken analyses of the FFF used in 3D printing process. However, only more recent studies have undertaken LCA of the FFF printing process. For example, Ma et al. [24] proposed positive solutions to reduce the environmental impact of FFF, and Faludi et al. [25] proposed an LCA based on machine utilization, to be applied to the comparison between FFF, Ink Jetting (IJ), and traditional manufacturing.

In a different way, the LCA method applied to PBF processes was the most extensively studied [1], since PBF is increasingly being used to manufacture products in industries including automotive and aerospace [5]. Selective Laser Sintering (SLS) and Selective Laser Melting (SLM) belong to this printing technology, and both technologies use laser beams to melt and solidify material into a powder bed according to layers of a corresponding three-dimensional (3D CAD) model [26,27].

Despite the excellent product properties produced via SLS, this method involves high upfront capital costs while mFFF is a cheaper process [9]. Moreover, as mentioned earlier, the mFFF process has the advantage of possibly being exported for production in microgravity or weightless environments, making this technology increasingly interesting for possible applications in future space missions, such as permanent remedy or temporary additive spatial repair [28]. Metal FFF is a candidate technology for MAM aboard the International Space Station [29].

This study is part of the results belonging to a research project aimed at developing a bio-based binder, mainly composed of polylactic acid (PLA), to produce Ti6Al4V feedstock suitable for MAM via mFFF [30]. The research project involved an analysis of the mechanical properties of a metal sample and a comparison of the mFFF process with other MEAM methods, such as SLS, in terms of environmental impact and energy consumption through the LCA method.

Therefore, the aim of the present paper is to show the results of the environmental comparison between two different MAM processes (SLS and mFFF) through LCA methods in order to manufacture the same metal sample. A similar comparative environmental impacts analysis of FFF and SLS technologies was carried out by Luo et al. [31] in 1997. More recently, a comparative LCA analysis between the FFF and SLS processes was researched by Tagliaferri et al. [32]. However, the compared materials were different (polymer vs. metals) and no comparative studies on the “Cradle-to-Gate” LCA of mFFF processes for the same metal sample are reported in scientific literature.

## 2. Materials and Methods

The Life Cycle Assessment (LCA) analysis method is a systematic approach used to evaluate the environmental impacts of a product, process, or system within its entire life cycle. The method involves assessing the inputs, outputs, and environmental effects at each stage of the life cycle, from raw material extraction to the final disposal.

LCA typically consists of four main steps [1]:

1. Goal and Scope Definition: Clearly defining the purpose and boundaries of the study, including the specific environmental impacts to be assessed and the functional unit of analysis.
2. Life Cycle Inventory (LCI): Collecting data on the energy, materials, and emissions associated with each life cycle stage, including resource extraction, manufacturing, transportation, use, and end-of-life management.
3. Impact Assessment: Evaluating the potential environmental impacts of the inputs and outputs identified in the LCI. This step involves applying impact assessment methods to quantify the effects on categories such as climate change, resource depletion, water pollution, and human health.
4. Interpretation: Analyzing and interpreting the results to draw conclusions about the environmental performance of the product, process, or system. This step involves identifying improvement opportunities and providing recommendations for reducing environmental impacts.

The following sub-sections illustrate the 4 steps applied to mFFF and SLS processes.

### 2.1. Goal and Scope Definition

The purpose of this analysis is the comparison, in terms of environmental impact, of two additive manufacturing methodologies, used for the printing of the same metal specimen. The analysis was carried out in accordance with the standard ISO 14,040 [33] and ISO 14,044 [34]. Furthermore, the Product Category Rules relating to metal products have been taken as a reference [35].

In more than 60 scientific papers examined by the work of Ruschi et al. [16] and published prior to 2019 regarding the LCA-AM binomial, 30 papers used the manufactured product as the functional unit, while in only 10 papers, the functional unit was the mass of the product.

If the study relates to a specific specimen, it is appropriate to use that object as a reference unit. However, when making comparisons, those performing the study must ensure functional equivalence between the objects evaluated [23].

Other studies examined [36,37,38,39] that relate to LCA applied to AM confirm that the realized object is, in any case, the functional unit if the analysis aims to compare two manufacturing technologies (conventional and/or additive) [40,41].

The functional unit used in this study is one sample (dimensions: length 75 mm, width 11.2 mm, and thickness 4 mm; volume: 3 cm^3^) of titanium alloy Ti6Al4V, made with the mFFF and SLS printing methods.

In a comparative LCA analysis such as in this study, a “Cradle-to-Gate” method is typically employed because the use and end-of-life stages have similar results [12].

**Figure 3:**
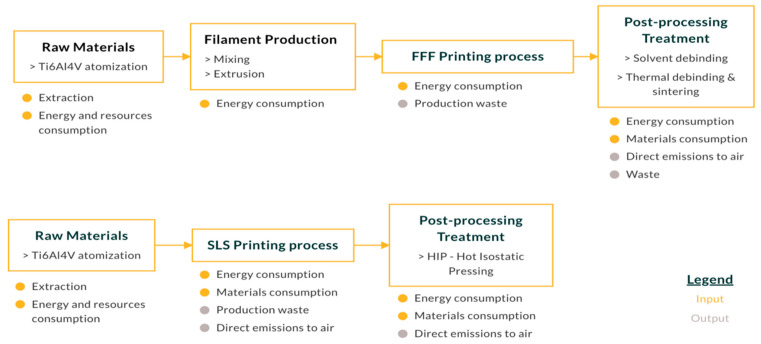
(Cradle-to-Gate) system boundaries.

Figure 3 shows the boundaries of the “Cradle-to-Gate” system used in this study.

Figure 3 shows the process from raw materials to post-processing treatment, and is divided into 3 main stages: pre-processing (raw materials—filament production or raw materials); printing process; and post-processing treatment. Within the boundaries of the system, the initial phase, such as CAD design, was not considered. Finally, the impact of the manufacturing of the printer used for the process was not considered.

The main steps of the mFFF and SLS processes are presented and described below.

#### 2.1.1. The mFFF Process

The mFFF process involves the following steps:

1. Heating the printer nozzle: The printer nozzle, also called the extruder, is heated to a specific temperature determined by the type of filament being used. The filament is fed into the nozzle in a molten state.
2. Printing the object: The printer starts the additive manufacturing process by moving the nozzle to the starting position on the build platform. The nozzle then begins extruding the molten filament onto the build platform, following the instructions from the sliced model. The filament is deposited layer by layer, solidifying as it cools.
3. Building layer by layer: The printer builds the object by repeating the process of extrusion and solidification for each layer. The build platform is incrementally lowered or the printer head is raised after each layer is completed, allowing the next layer of filament to be deposited on top of the previous layer.
4. Cooling and finishing: Once the entire object is printed, it is left to cool and solidify completely.
5. Post processing: Depending on the specific requirements of the object, additional post-processing steps may be performed to achieve the desired finish. In this case, solvent and thermal debinding and sintering is applied.

#### 2.1.2. The SLS Process

The SLS process involves the following steps:

1. Preparing the build platform: The SLS process starts by preparing the build platform, which is usually a flat surface or a removable tray. A thin layer of the powdered material, known as the build material, is evenly spread across the build platform.
2. Laser scanning and selective sintering: A high-powered laser beam is directed onto the powdered material, according to the specific cross-section of the object being manufactured. The laser scans and selectively sinters the powder particles together, solidifying them to form the first layer of the object.
3. Layer-by-layer building: Once the first layer is sintered, the build platform is lowered by a distance equal to the thickness of the next layer. A recoating mechanism then applies a fresh layer of powdered material on top of the previous layer.
4. Laser scanning and sintering (repeated): The laser scans and selectively sinters the new layer, fusing it with the previously solidified layer. This process is repeated layer by layer, with the laser scanning and sintering each new layer according to the digital design file.
5. Cooling and stabilization: After the entire object is built, it undergoes a cooling process to solidify and stabilize the structure. The build platform with the completed object is typically removed from the SLS machine.
6. Post-processing: Once the object has cooled down, it may require post-processing to improve its surface finish and mechanical properties. In this case, a Hot Isostatic Pressing (HIP) process is applied.

The choice of printing parameters, such as filament thickness and printing speed, were chosen to ensure the best mechanical performance of the sample [30], not considering environmental implications [24,42].

The modelling of the two 3D printing processes was carried out with the SimaPro 9.4.02 software. For the processes, where available, the Ecoinvent 3.8 datasets were used.

To calculate the impacts of the different emission contributions, the substances that contribute to an impact category were multiplied by a characterization factor that expresses the relative impact of the substance.

In order to evaluate the environmental impact of the printed sample in a complete perspective, the EPD method (2018) [35] was used. This method is used by the Environmental Product Declarations (EPDs) (Type III Environmental Declarations) and includes the following impact categories (Table 1).

### 2.2. Life Cycle Inventory (LCI)

According to the stages shown in Figure 3, this paragraph illustrates the LCI for each phase of both the SLS and mFFF processes: the raw material and filament fabrication; the printing process; and the post-processing treatment.

#### 2.2.1. Raw Material and Filament Fabrication

The raw material used for the production of the sample is the titanium alloy Ti6Al4V. This alloy is mainly composed of three chemical elements: titanium, aluminum, and vanadium, with some traces of other chemical elements such as iron, oxygen, and nitrogen. Within the aerospace field, the most used alloy is Ti6Al4V grade 5. Table 2 shows the weight percentage of each element in the titanium alloy used in this study.

The weight percentages in Table 2 were obtained as an average starting point from some data sheets and literature [43,44,45]. The titanium alloy, in order to be used in the mFFF and SLS processes, is previously atomized. Gas atomization is the conventional and more common process to obtain metal powder for Additive Manufacturing [45,46].

In this process, the metal is melted in a furnace and then atomized into droplets using high-pressure gas. These droplets are then directed into a cooling chamber filled with inert gas, where they rapidly solidify, forming metal powder particles with fine sizes (<100 μm), high sphericity, and excellent flowability [47,48,49].

**Figure 4:**
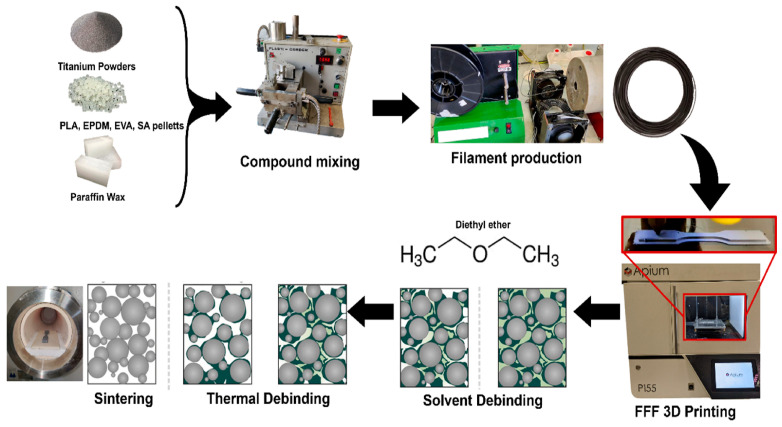
Schematization of the overall manufacturing process, i.e., compounding of raw materials for feedstock production; filament production through extrusion; mFFF 3D printing of specimen; solvent and thermal debinding; and consolidation through sintering to obtain the final metallic component [30].

In mFFF printing, a filament is used as the raw material. This consists of titanium powder mixed with polymeric binder, which is made from a well-defined mix of polymers [30]. The volume percentages of the raw materials used to produce the filament are reported in Table 3, and a schematization of the entire process from raw materials to a sintered sample is reported in Figure 4. In SLS printing, the powder is used without the production of further material.

#### 2.2.2. Printing Process

**Figure 5:**
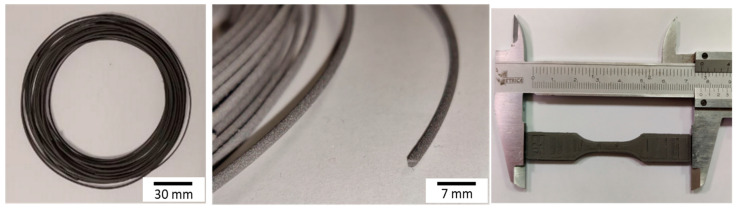
Extruded filaments and representative 3D-printed sample.

For the mFFF printing process:

The printing of the sample was carried out using the Apium P155 printer (Apium, in Karlsruhe, Germany), with a power of 40 W of the heater extruder and 150 W of the heater bed. The estimated consumption to produce a reference dog-bone ASTM D638-14 [50] Type V tensile sample (having the overall dimensions: length 75 mm, width 11.2 mm, and thickness 4 mm, corresponding to a volume of about 3 cm^3^) is 167 Wh, and a duration of 50 min. Titanium samples produced via the mFFF process have shown mechanical properties comparable with those of Metal Injection Molding (MIM) and SLS samples. After sintering, mFFF samples had a tensile yield strength of 662 MPa and an ultimate tensile strength of 742 MPa [30]. Figure 5 shows the extruded filament and examples of mFFF-printed specimens.

As brim support is not essential, production waste in the mFFF printing process can be avoided. In this study, no brim support is manufactured. Hence, no waste materials were produced.

For the SLS printing process:

The sample printing was modeled on the EOS Titanium Ti64 (EOSINT M 290) printer (EOS GmbH Electro Optical Systems, Munich; Germany), widely used for titanium alloys. Considering a power consumption of the printer of 3200 W and a printing speed of 5 mm^3^/s [49,51], the estimated consumption necessary for the production of the sample via SLS printing is 251 Wh.

According to a study by Kellens [52], for a similar printer (EOSINT P760, EOS GmbH Electro Optical Systems, Munich; Germany) the material wastage remained between 43.9% and 46.4% of the input material weight. According to [21], the waste powders of the printer (EOSINT M 290) are estimated to be around 50% of the powders used in the process. For the aim of this study, supported by research works [1,53], it has been assumed that material loss is approximately 5%, as liquid waste.

#### 2.2.3. Post-Process Treatment

For the mFFF post-process treatment:

The printed sample must undergo the debinding and sintering process. The sample is immersed in diethyl ether solvent in order to degrade part of the polymer for 24 h. This phase is called Solvent Debinding, where PW and SA (20% of the binder) are dissolved and disposed of as liquid waste. The heat treatment is then carried out in the oven. Upon reaching 550 °C, the polymer is degraded (thermal debinding), and there is a loss of polymeric mass as it becomes predominantly CO_2_ and water vapor [54].

The specimen is now brought to a temperature close to 90–95% of that of titanium fusion. The process takes place with a continuous flow of argon, under vacuum at −0.05 MPa, with an estimated consumption of 0.025 m^3^ of argon. Any post-sintering treatments such as sanding and surface finishing or coatings are not taken into account.

For the SLS process:

Specific treatments are necessary to achieve optimal mechanical properties due to the unique microstructure that originates from the SLS process. Hot Isostatic Pressing (HIP) is a widely employed thermomechanical post-processing technique aimed at reducing or eliminating internal defects in AM components, such as porosity or lack of fusion. As a result, the mechanical and fatigue properties of critical components are significantly enhanced [55].

Table 4 shows the consumptions derived from the HIP process [56], used for the modelling of the process.

Deboer et al. [36] reported an electricity consumption per kg of HIPed material equal to 3.95 kWh/kg, which is a lower value than the value used in this study. However, as no other information about this process is reported in the paper, the authors prudently decided to employ the data in Table 4.

### 2.3. Impact Assessment

In this section, LCA results are presented for the specimens made using both mFFF and SLS processes to identify the primary contributors to their environmental impact. The impacts are evaluated using the EPD (2018) method, with data sourced from the Ecoinvent database 3.8. Table 5 shows the values obtained for the assessment results.

The mFFF printing process has a higher overall impact, primarily due to the energy consumption necessary for producing a sample. In terms of kWh, the entire production process of the sample is much higher for the mFFF process, which has an energy consumption throughout its lifecycle of 7.31 kWh, compared to 0.32 kWh for the SLS process.

Furthermore, in filament production, a portion of plastic material is included, representing 45% of the filament’s volume. The most prevalent plastic material is PLA (25% of the sample’s volume), which has a high impact per kilogram, significantly affecting the Global Warming and Abiotic Depletion categories.

**Figure 6:**
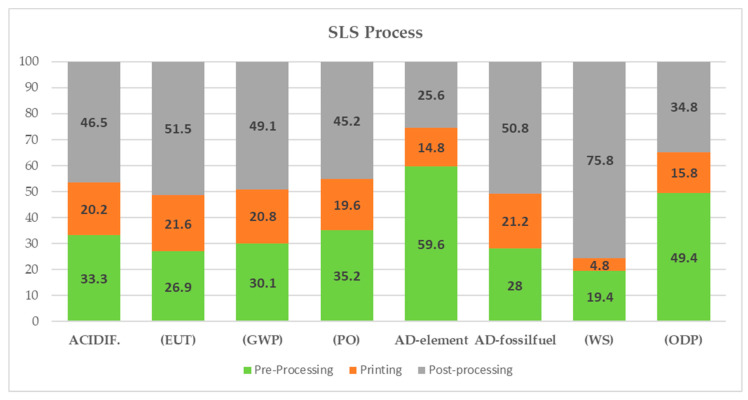
The SLS process’s impact distribution.

For the SLS process:

- The post-processing phase has the greatest impact in most categories, primarily due to the high consumption of argon required by the process (due to high temperatures and pressures). It represents approximately 98% of the process’s Global Warming Potential (GWP) impact. This phase varies in impact across categories, ranging from 34.8% for Ozone Depletion Potential (ODP) to 75.8% for Water Scarcity (WS), where it contributes the most. In this case, 99.7% of the impact is attributed to the use of argon, as a substantial amount of water is used for cooling during argon production;
- Powder atomization is the second most powerful contributor for most impact categories, mainly due to the presence of titanium, which has a high impact per kilogram and contributes significantly to all categories (69% of GWP, 73% of Abiotic Depletion Potential (ADP), and 65% of Eutrophication Potential (EUT)). Titanium represents the most significant contribution to ADP (93%). ADP measures the quantity of natural resources extracted and consumed throughout the product’s lifecycle and aligns with the results shown below;
- The printing phase has the least impact for all impact categories, ranging from 4.8% for WS to 21.7% for EUP. The SLS printing process’s impact distribution is shown in Figure 6.

**Figure 7:**
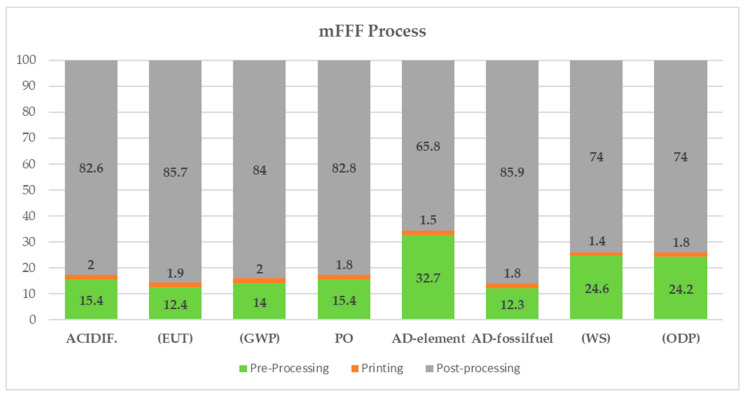
The mFFF printing process’s impact distribution.

For the mFFF process:

- The most powerful contribution pertains to the post-printing treatment process, which ranges from 65% for ADP to 86% for Abiotic Depletions (fossil fuels) and eutrophication. The major contribution of the process comes from the high consumption of electrical energy used in the process (6.5 kWh per sample) across all categories. In particular, electrical energy accounts for 89% of the Abiotic Depletions (AD) (fossil fuels) of the process, since AD (fossil fuels) comprises an impact category that measures the depletion of non-renewable resources, such as that caused by the production of electricity;
- Filament production represents the second most impactful contribution in terms of impact for most categories and varies from 12% for Abiotic Depletion (fossil fuels) to 32% for the Abiotic Depletions (elements) category. In this process, the highest impact is attributed to the atomization of powders used in filament production. As with the SLS printing process, the use of raw materials is particularly impactful in the ADP category, where the contribution related to powder atomization is 84%;
- The sample molding phase is the least impactful contribution, ranging from 1.4% for the WS (Water Scarcity) category to 2% for GWP and AD. The entire impact arises from the consumption of electrical energy used in the printing process. The mFFF printing process’s impact distribution is shown in Figure 7.

### 2.4. Interpretation

After having analyzed the consumption data for the entire lifecycle (Table 6) in detail, it is evident that the mFFF printing process is more energy-intensive compared to the SLS process. Furthermore, for both processes, the consumption primarily derives from the printing and post-treatment phases (94% mFFF; 88% SLS), while the extraction and preparation of raw materials phase has a relatively low impact on the overall energy consumption.

The CED (Cumulative Energy Demand) method allows for the assessment of the total amount of energy used throughout the entire lifecycle of a product or process, providing a comprehensive view of energy efficiency and potential opportunities for reducing the environmental impact associated with energy consumption. Energy is categorized into renewable and non-renewable, depending on the sources used.

The results obtained using the CED method are presented in Table 7.

## 3. Results and Discussion

**Figure 8:**
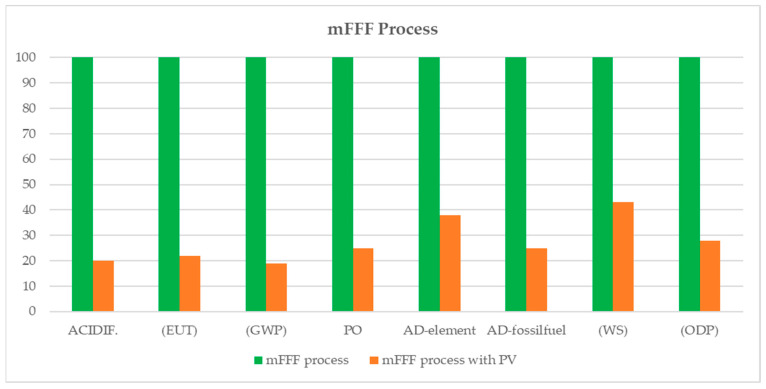
Comparison of the impact of mFFF printing with and without the use of photovoltaic electricity.

In order to assess the reduction in impact resulting from the use of renewable energy sources, an alternative scenario is proposed, in which the electrical energy used in the printing and post-process phases is exclusively generated from photovoltaic sources. The results show a significant reduction in impact, especially in the case of mFFF printing (Figure 8).

This is because the percentage contribution of electrical energy to the total impact of filament printing is high, and ranges from approximately 81% for GWP and Acidification to 56% for WS.

**Figure 9:**
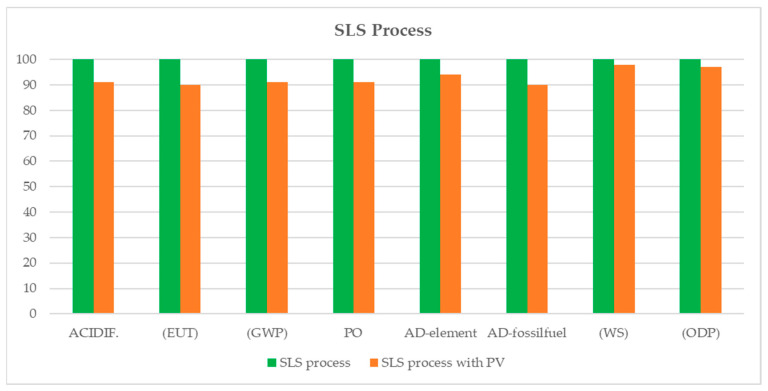
Comparison of the impact of SLS printing with and without the use of photovoltaic electricity.

For the SLS process the difference is less significant (Figure 9), since the percentage contribution of electricity to the total impact is smaller and varies from 21% for the Eutrophication category to 4% for WS.

The environmental impacts of the two types of additive manufacturing processes with the use of photovoltaic electricity are shown in the following table, Table 8.

When using photovoltaic electricity, the results show that the least impactful solution is filament printing. This is because electricity has a high contribution to the filament printing process, and therefore the respective overall reduction in impact is very high.

In order to delve deeper into the impact of the Global Warming Potential category, a comparison was made between the calculation method used for this indicator in the EPD (2018), i.e., the IPCC 2013 GWP100a, and the latest update of the method, i.e., the IPCC 2021 GWP100a. The comparison showed that the difference between the two impacts is almost nil, below 0.1%.

## 4. Conclusions

Recent studies [29] show how MEX appears to be gravity-independent, therefore it has been increasing attention for In-Space Additive Manufacturing (ISAM) due to its geometric freedom and reduced material waste, among other benefits. For this purpose, a bio-based binder composed mainly of polylactic acid (PLA) is being developed to produce Ti6Al4V feedstock suitable for use in metal Fused Filament Fabrication (mFFF), in order to manufacture a titanium alloy specimen for aerospace use [30]. The present study was carried out in order to compare the environmental impact of the mentioned specimen resulting from metal Fused Filament Fabrication (mFFF) and Selective Laser Sintering (SLS). Although comparative LCA analyses between FFF and SLS processes have been carried out in some studies—during which different materials were employed (polymer vs. metals)—no comparative studies on the “Cradle-to-Gate” LCA of mFFF processes for the same metal sample have been conducted. Using the EPD method (2018), the analysis considered the life cycle of the titanium alloy specimen starting from the pre-processing steps (raw materials and filament fabrication) and printing stage to the post-process treatments, excluding the use and the end-of-life phase of the specimen. The results from this method show a greater impact from the mFFF process in all the categories analyzed. The post-process phase generates the greatest impact for both the mFFF process and the SLS process. The impact of the mFFF process is due to both the high energy consumption and the disposal of solvents; for SLS, it is due to argon, which produces the 98% GWP of this phase.

For both processes, the least impactful phase is the printing stage, where the SLS process is most impactful mainly because its energy consumption is 1.5 times greater than that of mFFF and it generates 20% GWP and 21.7% EU, against 2% GWP and 1.9% EU in the mFFF process. In the pre-processing stage, titanium powder production contributes significantly to ADP (93% in SLS; 83% in mFFF) and GWP (69% in SLS; 45.6% in mFFF). As reported, relative to the printing and post-process phase, the use of electricity is the main determining factor in the different categories of impact. The same conclusion was reached following the analysis carried out with the CED method. Nevertheless, the situation changes if the needed electrical power is obtained from renewable energy sources.

An alternative scenario was proposed in which the electrical energy used in the printing and post-process phases is produced exclusively by photovoltaics. The results show that in this case SLS printing has more impact than mFFF printing, contrary to what was observed with the European energy mix. This study highlights how the reduction in impact derives from both manufacturing processes and energy sources. However, the difference obtained for the Global Warming Potential category using the updated IPCC 2021 GWP100a method is not significant.

In conclusion, although the mFFF process has been found to be less expensive than SLS, it is 50% more impactful. Under the examined conditions, the mFFF process is only more sustainable than the SLS process through the use of renewable energy. Further studies will focus on the comparison of the same processes in the production of larger samples (analyzing the scale factor) where brim supports are manufactured. This last aspect requires a greater deepening of understanding because although studies [57,58] show how PLA can be reused, the polymeric binder can change some properties, in particular the mechanical ones, through recycling cycles due to hydrolytic degradation. Therefore, the amount of material used as brim support could significantly affect the LCA results of the mFFF process.

## Figures and Tables

**Table 1:** Impact categories.

Impact Categories	Unit
Acidification	kg SO_2_ eq
Eutrophication	kg PO_4_^−3^ eq
Global Warming (GWP100a)	kg CO_2_ eq
Photochemical Oxidation	kg NMVOC
Abiotic Depletion, elements	kg Sb eq
Abiotic Depletion, fossil fuels	MJ
Ozone Layer Depletion (ODP)	kg CFC-11 eq
Water Scarcity	m^3^ eq

**Table 2:** Ti6Al4V composition.

Element	Weight %
Ti	89.8
Al	6.1
V	4.1

**Table 3:** Filament composition [30].

Raw Materials	Volume Percentage (%)
Ti6Al4V	55
PLA (polylactic acid)	25
EPDM (ethylene propylene diene monomer)	9
EVA (ethylene vinyl acetate)	2
PW (paraffin wax)	7
SA (stearic acid)	2

**Table 4:** HIP cycle consumptions [56].

HIP Cycle	Data
HIP parameters	
- Temperature (°C)	1.185
- Pressure (MPa)	150
- Hold time (h)	4
Electricity consumption (kWh/kg)	4.92
Gas consumption—argon (Nm^3^/kg)	0.23

**Table 5:** Impact assessment results.

Impact Categories	Unit	SLS Process	mFFF Process
Acidification	kg SO_2_ eq	5.20 × 10^−3^	1.42 × 10^−2^
Eutrophication (EUT)	kg PO_4_^−3^ eq	6.39 × 10^−4^	1.96 × 10^−3^
Global Warming (GWP100a)	kg CO_2_ eq	1.13	3.2
Photochemical Oxidation (PO)	kg NMVOC	2.63 × 10^−3^	7.40 × 10^−3^
Abiotic Depletion, elements	kg Sb eq	1.78 × 10^−7^	3.37 × 10^−7^
Abiotic Depletion, fossil fuels	MJ	1.25 × 10	3.90 × 10
Water Scarcity (WS)	m^3^ eq	1.31	1.05
Ozone Layer Depletion (ODP)	kg CFC-11 eq	6.87 × 10^−8^	1.49 × 10^−7^

**Table 6:** Electricity consumption for SLS and mFFF process.

**Additive Manufacturing Process**	**Total Electricity Consumption (kWh)**	**Electricity Consumption—Printing and Post-Treatment Phase (kWh)**
mFFF process	7.31	6.87
SLS process	0.32	0.28

**Table 7:** CED method results.

Impact Category	Unit	SLS	mFFF
Non-renewable, fossil	MJ	12.51	39.09
Non-renewable, nuclear	MJ	9.34	30.35
Non-renewable, biomass	MJ	0.00	0.00
Renewable, biomass	MJ	0.58	1.69
Renewable, wind, solar, geothermal	MJ	1.33	5.50
Renewable, water	MJ	1.87	5.61

**Table 8:** Impact assessment results with the use of photovoltaic energy.

Impact Categories	Unit	SLS Process	FFF Process
Acidification	kg SO_2_ eq	4.73 × 10^−3^	2.78 × 10^−3^
Eutrophication	kg PO_4_^−3^ eq	5.77 × 10^−4^	4.23 × 10^−4^
Global Warming (GWP100a)	kg CO_2_ eq	1.03	6.29 × 10^−1^
Photochemical Oxidation	kg NMVOC	2.41 × 10^−3^	1.86 × 10^−3^
Abiotic Depletion, elements	kg Sb eq	1.69 × 10^−7^	1.26 × 10^−7^
Abiotic Depletion, fossil fuels	MJ	1.13 × 10	9.84
Water Scarcity	m^3^ eq	1.29	4.58 × 10^−1^
Ozone Layer Depletion (ODP)	kg CFC-11 eq	6.43 × 10^−8^	4.11 × 10^−8^

## Data Availability

Data are contained within the article.

## Abbreviations

(ADP) Abiotic Depletion Potential; (AD) Abiotic Depletion; (AM) Additive Manufacturing; (BJ) Binder Jetting; (CAD) computer-aided design; (CC) climate change; (CFC-11) Trichlorofluoromethane; (DED) Directed Energy Deposition; (EPD) Environmental Product Declarations; (EPDM) Ethylene propylene diene monomer; (EUT) Eutrophication Potential; (EVA) Ethylene vinyl acetate; (FDM) Fused Deposition Modelling; (FFF) Fused Filament Fabrication; (HIP) Hot Isostatic Pressing; (LCA) Life Cycle Assessment; (LCI) Life Cycle Inventory; (ODP) Ozone Layer Depletion Potential; (GWP) Global Warming Potential; (MAM) Metal Additive Manufacturing; (MJ) Material Jetting; (ME, MEX, or MEAM) Material Extrusion Additive Manufacturing; (mFFF) metal Fused Filament Fabrication; (NMVOC) non-methane volatile organic compounds; (PBF) Powder Bed Fusion; (PO) Photochemical Oxidation; (SA) stearic acid; (SLM) Selective Laser Melting; (SLS) Selective Laser Sintering; (PLA) polylactic acid; (PW) paraffin wax; (WS) Water Scarcity.
